# Supplementary material for: The Systems Biology Research Tool: evolvable open-source software
Source: BMC Syst Biol. 2008 Jun 29;2:55. doi: 10.1186/1752-0509-2-55 (PMC2446383; doi:10.1186/1752-0509-2-55)
Supplement: Additional file 1 — SBRT Archive. An archive of the current version of the Systems Biology Research Tool. [file 1752-0509-2-55-S1.zip › sbrt-1.4.0/doc/developers_guide/api/sbrt/kernel/utilities/proc/package-summary.html]

sbrt.kernel.utilities.proc


|  |  |  |
| --- | --- | --- |
| **Overview** \| **Package** \| Class \| **Use** \| **Tree** \| **Deprecated** \| **Index** \| **Help** | |  |
| **PREV PACKAGE**   **NEXT PACKAGE** | **FRAMES**    **NO FRAMES**     **All Classes** |  |


---

## Package sbrt.kernel.utilities.proc

Provides classes and interfaces for performing utility processes.

**See:**
  
          **Description**

| **Class Summary** | |
| --- | --- |
| **IntervalComparison<V>** | This class is used to compare sets of intervals. |
| **VariableParticipation<E extends MathExpr<V>,V>** | This class is used to organize a collection of mathematical expressions according to the variables they contain. |
| **VectorComparison<V>** | This class is used to compare sets of intervals. |

## Package sbrt.kernel.utilities.proc Description

Provides classes and interfaces for performing utility processes.

---


|  |  |  |
| --- | --- | --- |
| **Overview** \| **Package** \| Class \| **Use** \| **Tree** \| **Deprecated** \| **Index** \| **Help** | |  |
| **PREV PACKAGE**   **NEXT PACKAGE** | **FRAMES**    **NO FRAMES**     **All Classes** |  |


---
